# The Use of Informed Consent in Endodontic Treatment in Saudi Arabia: A Cross-Sectional Study

**DOI:** 10.7759/cureus.60385

**Published:** 2024-05-15

**Authors:** Majed Almalki, Waad F Khayat

**Affiliations:** 1 Restorative Dentistry, College of Dentistry, Umm Al-Qura University, Makkah, SAU

**Keywords:** saudi arabia, informed consent, endodontic, dental ethics, autonomy

## Abstract

Introduction: Informed consent is a legal process intended to protect patients' rights and ensure ethical medical practice. The aim of this cross-sectional study was to assess dentists' practice of obtaining informed consent and adherence to the recommended guidelines, and to investigate the types of consent, barriers, the process, and the quality of the information included in preoperative informed consent for endodontic treatment in Saudi Arabia.

Methods: A validated self-administered online questionnaire was developed and distributed to dentists performing endodontic treatment in Saudi Arabia. Data were collected using a snowball sampling technique for three months (May to July).

Results: Of the 452 participants included in the study, 79.4% (n = 359) obtained informed consent, and 63.5% of them followed the recommended guidelines. Dentists with over 10 years of experience used informed consent regularly (p < .005). The main barrier to obtaining informed consent was the lack of a standardized format (28.5%). Furthermore, only 36.3% of the participants obtained written informed consent. Most participants (75.4%, n = 341) reported that they discuss and disclose information about the treatment to patients themselves. Less than 5% of participants include all listed criteria in their consent process.

Conclusion: Although obtaining informed consent prior to endodontic treatment was a broadly adopted process among the participants, their practice appears to be inadequate. Issues such as the type and quality of informed consent need to be improved through educational and awareness programs and strict implementation by responsible authorities.

## Introduction

Ethical and legal aspects of healthcare have become an integral part of high-quality dental practice across the world. Dental ethics is governed by a set of principles and values that include autonomy and respect for patients' self-governance [1,2]. One of the core ethical obligations for ensuring patients' right to make treatment decisions is the use of informed consent prior to dental treatment [2]. Informed consent was introduced in the dental field in the mid-1980s, and since then, the delivery of dental services has shifted toward a patient-centered model of practice [3].

A recent study showed that endodontics is the dental specialty with the third highest incidence of lawsuits [4]. Failure to inform patients about potential consequences, persistent pathology, or defective treatment is considered a form of malpractice [5]. The use of proper informed consent can reduce the likelihood of legal complaints. This is crucial, as inappropriate informed consent can lead to successful legal suits by patients even when the claims regarding negligence are weak [6,7].

The Saudi Commission for Health Specialties and the Saudi Ministry of Health (MOH) have developed guidelines for the informed consent process in the country, based on international standards, Saudi culture, and Islamic laws. These guidelines provide a general framework for good clinical practice informed by ethical standards [8,9].

To the best of the authors' knowledge, no previous dental studies in Saudi Arabia have investigated the types and quality of informed consent and the challenges in properly obtaining consent for endodontic treatments. Such information could help to identify key issues and concerns in the planning and development of well-structured consent forms to meet the quality requirements and accreditation standards to ensure effective and legal patient-centered endodontic practice. Therefore, the aim of the study was to assess dentists' practice of obtaining informed consent for endodontic treatment, their adherence to the Saudi Guidelines of Informed Consent, and the different types of consent, barriers, the process, and the quality of information included in preoperative informed consent for endodontic treatment in Saudi Arabia.

## Materials and methods

A brief explanation of this cross-sectional study was provided, and consent was obtained from all participants. The questionnaire was developed based on the Saudi Guidelines for Informed Consent and the guidelines of the American Association of Endodontics [9,10].

The electronic questionnaire was created in English. The content validity was confirmed by sending the questionnaire to 10 experts to evaluate the relevance and clarity of the questions. Reliability was checked using Cronbach’s alpha test, which reported an acceptable value of 0.8. A link to the questionnaire was distributed to target dentists via different social media platforms in May-July 2023 using a snowball sampling technique. Dentists who perform endodontic treatment in Saudi Arabia were included in this study. Undergraduate students and general dentists who do not perform endodontic treatment were excluded.

The questionnaire was divided into various sections. The first section determined eligibility to participate in the study. Participants who did not meet the inclusion criteria were excluded from the study. The following sections included questions regarding demographic data, frequency of using informed consent prior to endodontic procedures, awareness and implementation of the Saudi Guidelines of Informed Consent, barriers to their use, the process applied, the types of consent, and the details included in the endodontic consent.

The sample size was determined based on the number of healthcare workers in Saudi Arabia (2018-2027) [11]. A sample size of 351 was deemed adequate to obtain a 95% confidence level and a 5% margin of error for a population size of 5,829 (including licensed general dentists, postgraduate endodontic residents, and classified endodontists in Saudi Arabia). Data entry and analysis were done using Excel 365 (Microsoft Corporation, Redmond, Washington) and IBM SPSS Statistics for Windows, Version 28 (Released 2021; IBM Corp., Armonk, New York). Counts and percentages were reported. Chi-square tests, post-hoc tests, and pairwise comparisons of proportions were applied for statistical analysis.

## Results

A total of 452 participants were included in the study. The counts and percentages of participants based on gender, experience, workplace, and clinical rank are presented in Table 1. The frequency of the participants’ use of endodontic informed consent is shown in Table 2. A chi-square test was performed to identify associations between the practice of obtaining informed consent in endodontics and the demographic variables. Level of experience (X² = 43.8, p < .001) was significantly associated with the use of informed consent in endodontics. Dentists with more than 10 years of experience used informed consent regularly in endodontics practice (p < .005). No significant influence on obtaining informed consent was found for gender (X² = 0.7, p = 0.69), workplace (X² = 12.1, p = 0.06), or clinical rank (Χ² = 9.3, p = 0.06).

**Table 1:** Descriptive data of variables of interest.

Variables of Interests		n	%
Gender	Male	237	52.4
	Female	215	47.6
Experience	Less than 5 Years	190	42.0
	5–10 Years	137	30.3
	More than 10 Years	125	27.7
Workplace sector	Academic Healthcare	44	9.7
	Governmental Healthcare	136	30.1
	Military Healthcare	112	24.8
	Private Healthcare	160	35.4
Clinical rank	General Dentist	225	49.8
	Postgraduate Resident	64	14.1
	Specialist/Consultant	163	36.1

**Table 2:** Frequency of obtaining informed consent among participants.

Frequency of obtaining informed consent in endodontic treatment		n	%
Always (regularly obtain consent in all endodontic cases)	188		41.6
Sometimes (less regularly obtain consent or in some cases)	171		37.8
Never or rarely obtain consent	93		20.6
Total	452		100

**Figure 1:**
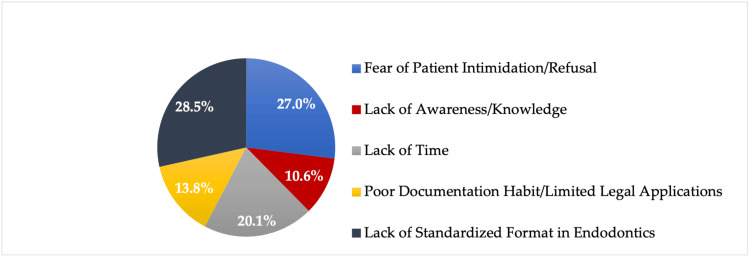
Barriers and challenges that influence the use of informed consent in endodontic treatment (%).

Information about the implementation of Saudi Guidelines for Informed Consent among the participants who reported using informed consent is shown in Table 3. Following the recommended guidelines was significantly associated with the participants’ level of experience (X² = 34.5, p < .001), workplace (X² = 46.3, p = .006), and clinical rank (X² = 18.2, p = .006). Dentists with more than 10 years of experience (p = .004) and those working in the private healthcare sector (p = .003) were likely to follow the recommended guidelines, whereas dentists working in the military healthcare sector tended to follow different guidelines regarding informed consent (p < .001). Postgraduate endodontic residents were largely unaware or unsure of following the Saudi Guidelines of Informed Consent (p = .001). The various barriers that influence the use of informed consent in endodontic practice are shown in Figure 1.

**Table 3:** Implementation of guidelines recommended by the Saudi Guidelines for Informed Consent among participants who reported using informed consent.

Frequency of following the guideline		n	%
Participants following Saudi Guidelines for Informed Consent	228		63.5
Participants following other guidelines	83		23.1
Participants were not aware/not sure which guideline was followed	48		13.4
Total	359		100

**Figure 2:**
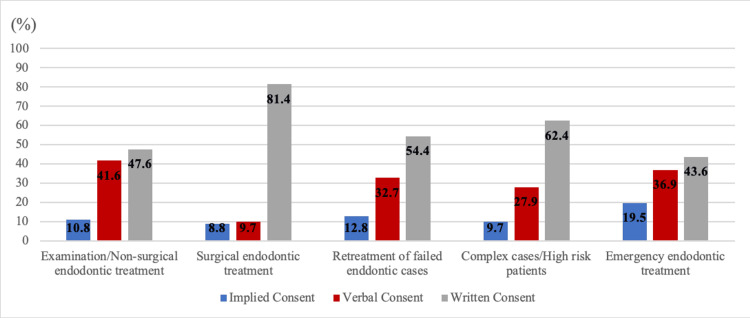
Types of informed consent obtained for various endodontic cases (%).

The study also investigated the types of informed consent obtained in endodontic treatment. The frequencies with which the participants use the various types of informed consent are shown in Table 4, while the percentages are presented in Figure 2.

**Table 4:** Frequency of using various types of informed consent in endodontic treatment among participants who reported using informed consent.

Type of informed consent		n	%
Written and signed informed consent	130		36.3
Mix of verbal and written consent	195		54.3
Verbal consent	16		4.4
Implied consent	18		5.0
Total	359		100

Information about the process of obtaining informed consent for endodontic treatment was collected from the participating dentists. Based on the results, 75.4% (n = 341) of the dentists discuss and disclose information about the treatment to patients by themselves before the treatment, while 11.1% (n = 50) stated that such information is given to the patients by an assistant or a staff member. In addition, 10.4% (n = 47) of the participants reported that patients receive information about the treatment and procedures through various education resources by reading or watching videos. The written consent forms are most commonly signed by both the dentist and the patient/guardian (37.6%, n = 170). However, 30.9% (n = 140) of the participants indicated that consent forms are only signed by patients/guardians, while 16.3% (n = 74) stated that a witness signed as well. Most of the dentists (79.6%, n = 360) indicated that the minimum age to provide informed consent was 18 years. Regarding the valid duration of consent, 34.7% (n = 157) of the dentists reported that it is considered valid until the end of the specified treatment, 10% (n = 45) stated it is valid for 30 days, while 30.8% (n = 139) did not specify the duration.

The quality of informed consent was evaluated by exploring the content, information, and details included in the process of obtaining informed consent prior to endodontic treatment as well as the confirmation of the patient’s understanding of the information (Table 5). Less than 5% of the participants reported that they include all of the listed criteria in their consent process.

**Table 5:** Type of information and details disclosed when obtaining informed consent before endodontic treatment.

Information	Details disclosed during endodontic informed consent	n	%
Complications	Common complications (swelling, bleeding, infection, pain, and sensitivity)	321	71.0
	Transient/permanent numbness/tingling sensation in the oral tissue	200	44.2
	Loosening of crown/bridge	133	29.4
	Delayed healing	129	28.5
	Allergic reaction to anesthesia/medication/materials	121	26.8
	Face/tooth discoloration	93	20.6
	Jaw muscle spasm/temporomandibular disorders	87	19.2
	Referred pain to head, neck, or ear	86	19.0
	Changes in occlusion	48	10.6
Risks	Risk of treatment failure	246	54.5
	Risk of broken instruments/perforation of root or sinus	239	52.9
	The risks and frequent exposure to X-rays during the treatment	82	18.1
	Details of the existing condition	63	13.9
	Side effects of medications, including drowsiness or lack of coordination	39	8.6
	The influence of antibiotics on the effectiveness of birth control bills	39	8.6
Treatment and outcome	All possible alternative treatments, benefits, risks, and cost-effectiveness	118	26.1
	The option of “no treatment” and consequences	66	14.6
	Details of the procedures, visits, and post-operative care	127	28.1
	The need for restorative treatment after endodontic treatment	100	22.1
Legal	A statement of patient’s understanding of the case and the answers to questions	169	37.4
None	No specific information given/no consent before endodontic treatment	97	21.5

## Discussion

In Saudi Arabia, according to Article 19 of the Law of Practicing Healthcare Professions, it is prohibited to perform medical interventions before obtaining informed consent from patients [12]. Recently, the Ministry of Health (MOH) published guidelines regarding informed consent in Saudi Arabia to raise awareness among practitioners and the community about informed consent and to unify and govern the process and quality of informed consent [9].

In the present study, the majority of the respondents (79.4%) claimed that they obtain informed consent prior to endodontic treatments. Of these, 41.6% stated that they always obtain consent, while 37.8% indicated they obtain consent less regularly. A previous survey conducted in Turkey found that 63% of participants obtained informed consent before endodontic treatment [4].

The most frequently reported barrier to obtaining informed consent among the participants was the lack of a standardized format for endodontic treatment (28.5%). Although the Saudi guidelines hold clinicians responsible for obtaining and documenting informed consent, the awareness, development, and implementation of informed consent policies are the responsibility of the organization’s management team [9]. Fear of patient intimidation was the second most common barrier to obtaining informed consent, similar to a report from a previous study on medical surgeons in Saudi Arabia [13].

In this study, 63.5% of the participants claimed that they follow the Saudi Guidelines of Informed Consent. Furthermore, the statistical analysis revealed a significant correlation between more than 10 years of experience and adherence to the guidelines. Dentists with extensive experience may have a greater understanding of patients’ rights and associated legal risks due to their exposure to clinical practice and patient complaints. Furthermore, dentists working in the private sector commonly followed the guidelines. The private sector in Saudi Arabia is monitored and audited by the MOH dental licensing offices, and it has been speculated that this positively impacts adherence to regulations due to their repeated visits to these clinics [14].

While the MOH regulates and supervises all healthcare sectors in Saudi Arabia, respondents from the military healthcare sector generally follow different guidelines for informed consent. Moreover, postgraduate endodontic residents were significantly unaware of the guidelines that should be followed. These findings appear to confirm the reported lack of MOH authority over the military and academic healthcare sectors [15].

The Saudi Guidelines of Informed Consent recommend obtaining signed written informed consent for any treatment that carries a potential risk of side effects, such as surgeries, interventions, hospital admission, and the use of anesthesia or sedation. In addition, a patient’s silence should not be considered consent [9]. Similar regulations are applied in other countries, including the UK, the United States, and Australia [10,16,17]. However, among the participants who stated that they obtain informed consent, only 36.3% obtained written and signed informed consent, while 54.3% obtained a mix of verbal and written consent, 4.4% obtained only verbal consent, and 5% obtained implied consent. In contrast, a Turkish study revealed that 74.5% of dentists obtained written consent, while 25.5% obtained verbal consent before endodontic procedures [4]. The low adherence to obtaining written consent in our study may be attributed to a lack of organizational rules or strict implementation of the recommended guidelines, as evidenced by the fact that the absence of a standardized consent format was reported as a barrier by the participants.

Regarding the type of informed consent obtained, written informed consent was the most common type in all endodontic procedures, especially endodontic surgical procedures (81.4%). The highest reported use of implied consent was for emergency treatment (19.5%), while verbal consent was more common for non-surgical root canal treatment, examination, and diagnosis (41.6%). The participants showed a trend of seeking more protection when the procedure carries a higher level of complexity and risk, reflecting the results of previous studies [18,19].

Although signing an informed consent verifies the patient’s consent for treatment, it is only considered valid when it has been discussed by the clinician [16]. The majority of our participants discuss and disclose information with patients themselves (75.4%), although a small percentage fulfilled the requirement of obtaining signatures from the patient, the dentist, and a witness.

One of the important elements in the governance of informed consent is the validity period of the form. The validity period must be specified, and it should be no longer than 30 days from the date of signature [9]. However, only 10% of the participants considered a consent form valid for 30 days, and the rest either did not specify the validity period or considered consent to be valid until the end of the specified treatment.

The minimum age for patients to sign an informed consent form varies depending on the laws in a country. In Saudi Arabia, the minimum age for the patient to sign an informed consent form is 18 years. Similarly, in the United States, patients under the age of 18 years cannot provide informed consent, and parents or legal guardians must do so instead [10]. However, according to the European Union Agency for Fundamental Rights, there are inconsistencies between European countries regarding the minimum age of consenting to medical treatment [20]. In our study, most of the participants (79.6%) were aware of the minimum age for treatment consent in Saudi Arabia.

While endodontic treatment has a high success rate, it is the second most common treatment type reported in malpractice litigation cases in Saudi Arabia [21,22]. Therefore, clinicians must disclose the appropriate information for each procedure based on the recommended guidelines to avoid possible legal consequences. Nevertheless, our findings suggest discrepancies in the information disclosed to patients. While most of the participants claimed that they disclosed common complications and risks, such as infection, swelling, pain, risk of treatment failure, and risk of separated instrument or perforation, other complications and risks, such as numbness, loss of a crown, an allergic reaction, discoloration of the face or tooth, jaw muscle spasm, referred pain, changes in occlusion, side effects of medication, and the risk of frequent exposure to X-rays, were not commonly disclosed to the patient. Furthermore, most of the participants indicated they did not disclose information about treatment outcomes or alternatives, including information regarding treatment, details of the procedure, or a restorative treatment plan.

The limitations of the present study include the possibility of selection bias, which may occur in self-administered survey-based studies, and sampling bias, which can result from the use of convenience snowball sampling. Caution should be used in generalizing the results to dentists and dental healthcare systems using different types of guidelines. As no similar previous studies investigating the quality and process of endodontic informed consent have been conducted in Saudi Arabia, the design of this study was adequate to provide preliminary data for informing the design of further comprehensive studies.

## Conclusions

This study highlights the current status of endodontic informed consent practice among dentists in Saudi Arabia. While most of the participants reported that they obtained informed consent prior to endodontic treatment, their practice appears to be inadequate. Furthermore, the lack of a standardized format and fear of patient intimidation were the most common barriers to obtaining consent.

Moreover, adherence to the recommended guidelines regarding the type, validity, and content of consent needs to be improved through enhanced organizational management, strict implementation of the regulation, and the implementation of educational and awareness programs in all healthcare sectors in Saudi Arabia.

## Appendices

Survey questionnaire used to generate the electronic survey is given in Table 6.

**Table 6:** Survey questionnaire used to generate the electronic survey.

Questions
1. Do you perform endodontic treatment currently in Saudi Arabia?
Yes
No
2.What is your clinical rank?
Specialist/consultant
General dentist
Resident in postgraduate specialty program
Undergraduate dental student
3. Please provide your gender:
Male
Female
4. How many years of experience you have in endodontics?
Less than five years
Five to ten years
More than ten years
5. Choose the sector in Saudi Arabia that you mainly work in? (if you work in more than one sector, please choose the main sector in which you practice most endodontics):
Governmental healthcare sector (Ministry of Health)
Private healthcare sector
Private academic sector
Governmental academic sector
Armed Forces healthcare sector
National guard healthcare sector
Other:
6. How frequently do you use informed consent in endodontic treatment?
Always (regularly obtaining informed consent in all endodontic cases)
Sometimes (less regularly obtaining informed consent or in some cases)
Never or rarely obtaining informed consent in endodontic cases
7. Do you follow the guideline/design of the dental informed consent recommended by the Saudi Ministry of Health (MOH)?
I follow MOH guidelines of informed consent
I follow different guidelines of informed consent
I am not aware/not sure which guideline is followed
8. What type of informed consent do you obtain routinely for each of the following type of endodontic case/treatment? (You can choose more than one if applicable for each case)
Non-surgical root canal treatment
Written consent
Verbal consent
Implied consent
Surgical root canal treatment
Written consent
Verbal consent
Implied consent
Cases of retreatment/previously initiated by others/failed
Written consent
Verbal consent
Implied consent
High-risk/medically compromised/complex cases
Written consent
Verbal consent
Implied consent
Emergency endodontic cases
Written consent
Verbal consent
Implied consent
9. What is the most significant barrier for using informed consent in endodontic clinical practice?
Fear of patients intimidation/ refusal
Lack of awareness/knowledge
Lack of time
Poor documentation habit/Limited legal application
Lack of standardized form in endodontics
Other:
10. Who mainly carries out the discussion of the consent form with the patient/guardian?
Dentist
Dental assistant/staff member
Providing educational resources to patients (reading/watching videos)
Not sure
11. Who signs the written consent form? (You can choose more than one if applicable)
The patient/guardian
The dentist
A witness
Not sure
12. What is the minimum age of patients to give a consent by themselves on dental treatment?
16
18
21
Other:
13. What is the validity period of the informed consent form?
30 days
Until treatment is completed
Not specified in the form
Not sure
Other:
14. Which of the following are included into the informed consent form that you currently use (You can choose more than one if applicable)?
Complications such as swelling, bleeding, infection and pain/sensitivity
Transient/permanent numbness and/or tingling sensation in the oral tissues
Jaw muscle spasm/ TMJ disorder
Changes in occlusion
Loosening of crowns/ bridge
Allergic reaction to anesthesia/ medications/ materials
Referred pain to ear/ head/ neck
Delayed healing
Risk of treatment failure
Risk of broken instruments/ perforation of root/ sinus
Face/ tooth discoloration
Side effects of medications causing drowsiness and lack of coordination
The influence of antibiotics on effectiveness of birth control bills
Details of the existing condition
All possible alternative treatment
The option of "no treatment"
Details of the procedures and consequences
The need for restorative treatment after root canal treatment
The risk and frequent exposure to X-rays during treatment
A statement of confirmation that patients understand the condition, treatment and answers to all their questions
No consent/specific information given to patients before endodontic treatment
Others:
